# Supplementary material for: A Buckycatcher in Solution—A Computational Perspective
Source: Molecules. 2023 Mar 21;28(6):2841. doi: 10.3390/molecules28062841 (PMC10056437; doi:10.3390/molecules28062841)
Supplement: Supplementary file 1 [file molecules-28-02841-s001.zip › molecules-2274800-supplementary.pdf]

A Buckycatcher in Solution—A Computational Perspective

Supporting Information

Filipe Menezes * and Grzegorz M. Popowicz *

Institute of Structural Biology, Helmholtz Zentrum Muenchen, Ingolstädter Landstr. 1, 85764 Neuherberg, Germany
* Correspondence: filipe.menezes@helmholtz-muenchen.de (F.M.); grzegorz.popowicz@helmholtz-muenchen.de (G.M.P.)

Generation Initial Structures

Initial geometries for the pincer in the ”main” open conformation were taken directly from the S12L database [68], by removing the fullerene molecules. Starting geometries for other conformers were obtained by modifying the S12L geometry of the main conformer in Avogadro [69,70] followed by restricted Universal Force Field (UFF) optimization to relax the structures. For the transition states connecting the different conformers of the pincers we took advantage of the fact that the UFF optimization in Avogadro generated planar arms for the corannulene pincer. This yielded very good starting geometries for the saddle points.
Initial geometries for the transition states associated with free tetrachloroethane were assembled manually in Avogadro [69,70]. After optimization of the free transition states, these were introduced in between the catcher's arms.

Description of Adducts with Solvent Molecules

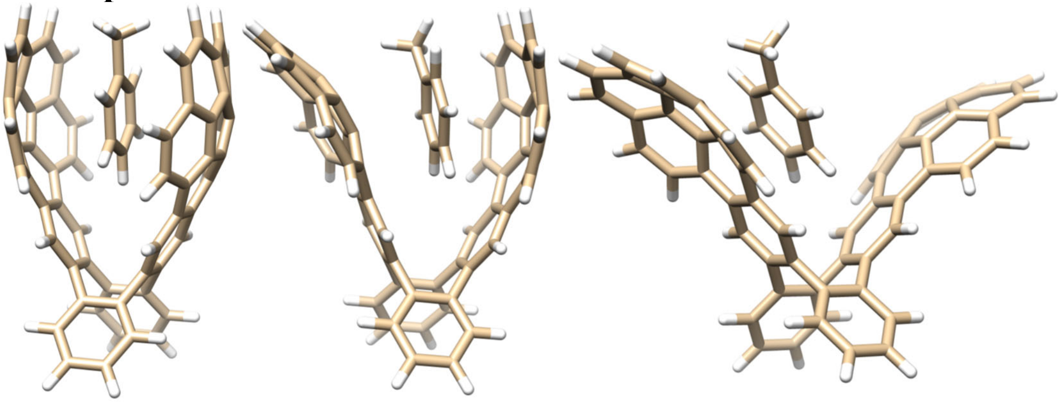

Figure S1: Caged adducts between the buckycatcher and toluene.

In the study of the conformational equilibria of $C_{60}H_{28}$ in toluene we also considered the explicit formation of aggregates between the corannulene pincers and solvent. Here we looked at several binding modes and configurations, with a particular focus on aggregates in which one molecule of toluene sits inside the buckycatcher. Snapshots of these complexes are given in Figure S1. In a second instance we considered the addition of two molecules of toluene inside each pincer's arms, as well as attachment of toluene to the outer surfaces of $C_{60}H_{28}$ conformers. We surveyed furthermore the aggregation of three molecules of toluene to each catcher, in this case however we only considered cases with one molecule of toluene in between the pincer's arms. In all cases contemplated, aggregation of toluene leads to complexes with no symmetry, except for the adduct with one toluene on the *i* face of conformer *iet*. For instance, when toluene is within the pincer's arms, the methyl group of toluene leads to asymmetry as

toluene tries to accommodate itself in a way that minimizes repulsive interactions. When toluene is attached to the outside of the buckycatcher, two types of aggregation were observed depending on the arm's curvature. In both cases, the aromatic ring of the solvent tries to attach itself to the flattest region of the pincer's arms, and the accommodation of the methyl group will determine the final symmetry. Besides complexes in which toluene is caged by the buckycatcher, we optimized adducts in which toluene π-stacks on one of the pincer's arms and forms a T-shape type of interaction with the base of the other. We could thus far only optimize such a stationary point at the GFN2-xTB/ALPB level in polar media (*e.g.* acetonitrile). Similar complexes were readily optimized using PM6-D3H4X/COSMO with either dielectric constant mentioned in the computational details.

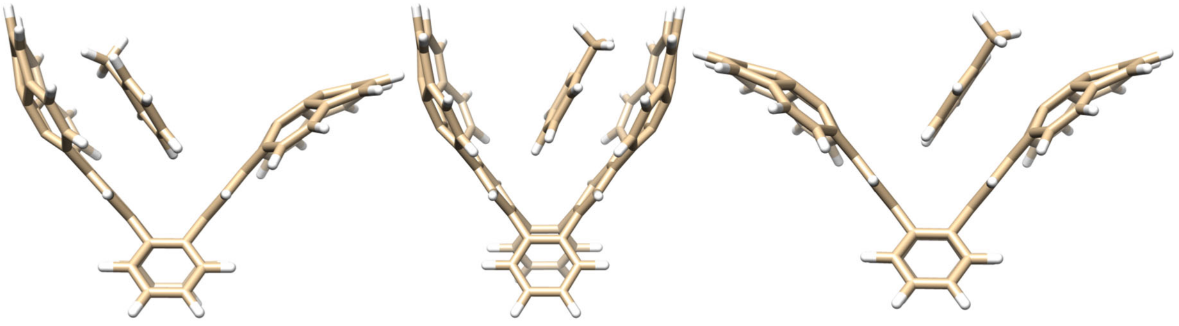

Figure S2: Structure of the adducts between the buckycatcher and toluene with differential interaction with the pincer's arms.

We also studied the encapsulation of other solvents in *ii*'s arms, namely chloroform, dichloromethane, 1,1-2,2-tetrachloroethane, acetonitrile and n-hexane. For tetrachloroethane we considered two possible conformations, a more symmetric one with pairs of chlorine atoms anti with respect to each other, and a less symmetric conformer obtained by rotating around the C−C bond by 60°. In the complex with acetonitrile the nitrogen atom of the solvent is pointing towards the tethers, with acetonitrile adopting a straight posture within the cage. The symmetry of the adduct is C_2. In the case of chloroform, though we started with an initial geometry in which each arm of the pincer would be in contact with chlorine atoms, the structure rearranged during geometry optimization so that all chlorine atoms point towards the same arm of the pincer. The chlorines position themselves in a way that the final complex has no symmetry. A similar case took place with dichloromethane, in which the starting geometry underwent a strong rearrangement in order to place the chlorine atoms in a way that minimizes repulsion with the pincer's arms, while allowing the latter to close as much as possible. This complex has symmetry C_S as well. Complexes with tetrachloroethane were particularly interesting because besides repulsive forces between solvent and pincer there are also stereo effects to consider from the solvent molecule itself. Thus, the least symmetric form of the solvent reoriented itself inside $C_{60}H_{28}$ in a way that one of the chlorine atoms pushes one arm of the pincer outwards. This makes the asymmetry in the adduct even more pronounced because the former comes from both molecules and not from the way the pair interacts. For the most symmetric isomer of tetrachloroethane the pincer can close its arms tighter around the solvent molecule. The C_i symmetry from the solvent is however lost, with the final adduct also asymmetric. In the case of hexane, due to the molecule's size, an ethyl group is placed outside the cage formed by the corannulene pincer. In static studies we considered only the linear form of n-hexane and the molecule places itself in the pincer's arms such that the final complex has a single plane of symmetry (point group C_S).

Adducts between the solvents above mentioned and the other conformers of the catcher were also optimized at the GFN2-xTB level. The complexes between chloroform and *ee* or *ii* are identical to what was described in the previous paragraph for *ii*. The symmetry in the case $CHCl_3$@*ie* is C_S. In the case of $CHCl_3$@*ee*, chloroform has more freedom, and the minimum of energy has no symmetry. In the case of dichloromethane, the molecule adopts in the complexes with *ee* and *ie* orientations different from the case with *ii*. While in CH_2Cl_2@*ee* the chlorine atoms lie really in between the pincer's arms, in the case of CH_2Cl_2@*ie* one of the chlorine atoms points to the *i* arm of the catcher. The symmetries in both cases are C_1 because in CH_2Cl_2@*ee* dichloromethane is tilted. Complexes with acetonitrile are in both cases C_S. The solvent molecule is however not perfectly parallel to the pincer's arms. In the complex with *ee* acetonitrile sits in a way the triple bond lies along the valley formed by the pincer's arms. The complex of hexane with *ee* has C_S symmetry, whereas the adduct with *ie* has symmetry C_1. These complexes are reasonably similar to the complex with *ii*.

Conformation equilibria according to PM6-D3H4X in gas phase

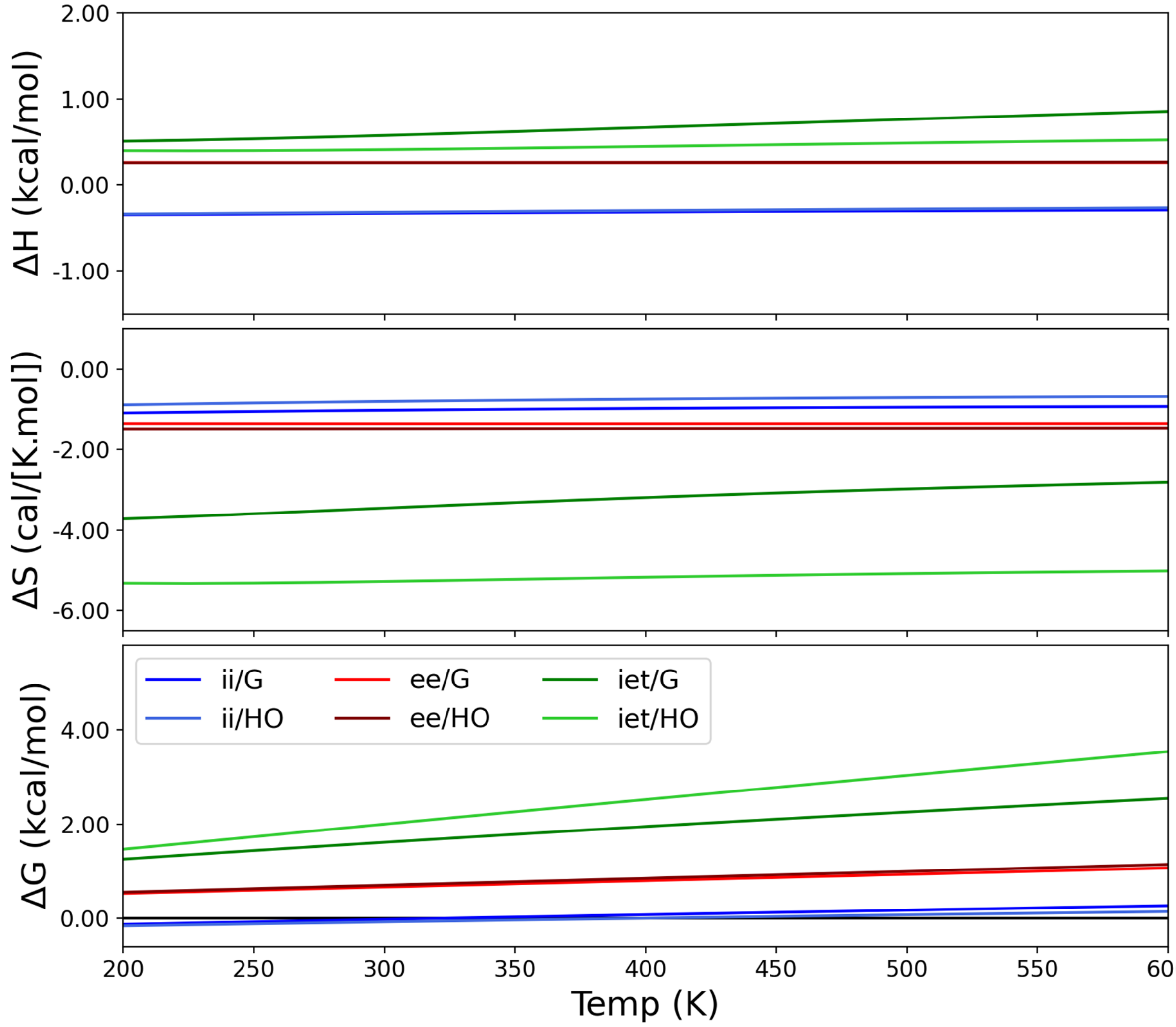

Figure S3: Thermodynamic functions for the corannulene pincer conformers with respect to species *ie*. Data generated from the PM6-D3H4X calculations.

Thermodynamics for second set of complexes between toluene and the Buckycatcher

Table S1: Binding energies, enthalpies (gas), entropies (gas) and Gibbs free energies (gas and in toluene) for the formation of aggregates between the conformers of $C_{60}H_{28}$ corannulene pincer with toluene, in which the latter establishes different interactions with each of the pincer's arms. Thermodynamic data is calculated at 300 K, all energies are in units of kcal/mol, whereas entropies are in units of cal.K^{-1}.mol^{-1}.

	PhMe@*ii*	PhMe@*ie*	PhMe@*ee*
ΔE_{bind}^{GFN2}	13.37	13.59	11.97
$\Delta E_{bind}^{PM6-D3H4X}$	12.95	12.82	9.71
$\Delta E_{bind}^{PM6-D3H+}$	12.79	12.86	9.91
ΔH_{gas}^{GFN2}	-13.40	-13.41	-12.26
$\Delta H_{gas}^{PM6-D3H4X}$	-12.02	-11.96	-9.02
$\Delta H_{gas}^{PM6-D3H+}$	-11.81	-11.96	-9.17
ΔS_{gas}^{GFN2}	-41.04	-43.87	-39.42
$\Delta S_{gas}^{PM6-D3H4X}$	-46.02	-45.75	-43.52
$\Delta S_{gas}^{PM6-D3H+}$	-45.93	-45.65	-43.43
ΔG_{gas}^{GFN2}	-1.09	-0.25	-0.43
$\Delta G_{gas}^{PM6-D3H4X}$	1.79	1.77	4.03
$\Delta G_{gas}^{PM6-D3H+}$	1.97	1.73	3.86
ΔG_{PhMe}^{GFN2}	2.42	3.10	1.52
$\Delta G_{PhMe}^{PM6-D3H4X}$	1.85	3.07	5.60
$\Delta G_{PhMe}^{PM6-D3H+}$	2.03	3.04	5.43

Thermodynamics for aggregation of two and three molecules of toluene

Below the thermodynamic data pertaining the aggregation of two and three molecules of toluene to the buckycatcher's conformers. All thermodynamic data was built based on the reactions

$$PhMe \; + \; (n-1)PhMe{:}A \; \rightleftharpoons \; (n)PhMe{:}A$$

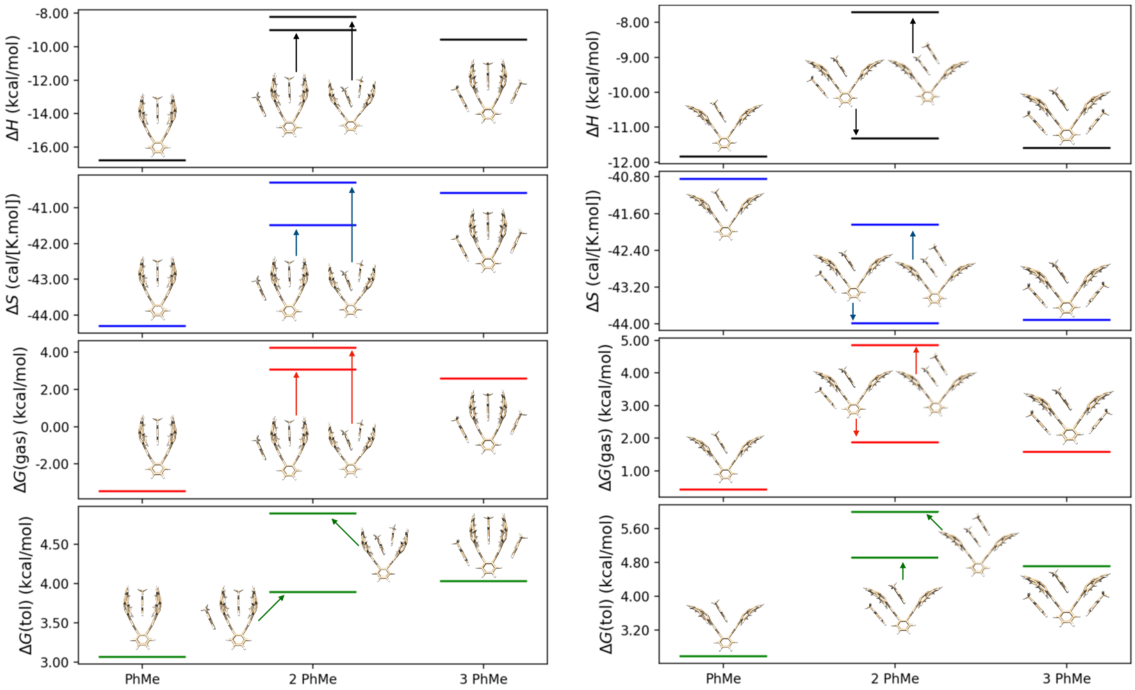

Figure S4: Thermodynamic data for adding two and three molecules of toluene for the conformers *ii* and *ee*.

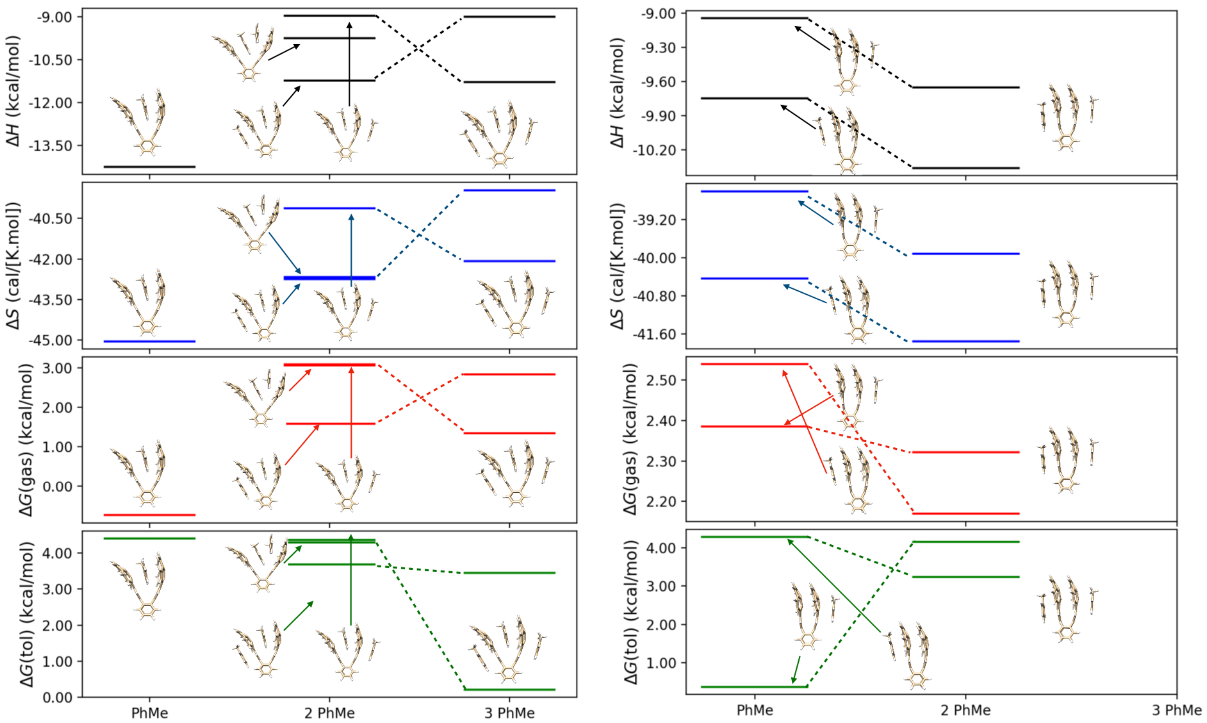

Figure S5: Thermodynamic data for adding two and three molecules of toluene for the conformers *ie* and *iet*.
